# Existential decision-making in a fatal progressive disease: how much do legal and medical frameworks matter?

**DOI:** 10.1186/s12904-017-0252-6

**Published:** 2017-12-28

**Authors:** Christian Weber, Barbara Fijalkowska, Katarzyna Ciecwierska, Anna Lindblad, Gisela Badura-Lotter, Peter M. Andersen, Magdalena Kuźma-Kozakiewicz, Albert C. Ludolph, Dorothée Lulé, Tomasz Pasierski, Niels Lynöe

**Affiliations:** 1 Institute of the History, Philosophy and Ethics of Medicine, University of Ulm, Parkstraße 11, 89073 Ulm, Germany; 2 Institute Józefa Piłsudskiego Warszawie, University of Warsaw, Marymoncka 34, 00-968 Warsaw, Poland; 3 Department of Neurology, Warszawski Uniwersytet Medyczny, Medical University of Warsaw, ul. Żwirki i Wigury 61, 02-091 Warsaw, Poland; 4 Department of Learning, Informatics, Management and Ethics, Karolinska Institute, Stockholm, Tomtebodavägen 18, 171 77 Solna, Sweden; 5 Department of Pharmacology and Clinical Neuroscience, Umeå University, -90187 Umeå, SE Sweden; 6 Department of Neurology, University of Ulm, Oberer Eselsberg 45, 89081 Ulm, Germany; 7 Department of Ethics, Center for Bioethics and Biolaw, University of Warsaw, Institute of Philosophy, Krakowskie Przedmieście 3, 00-927 Warsaw, Poland

**Keywords:** Amyotrophic lateral sclerosis (ALS), Shared decision-making, Therapeutic treatment, Palliative care, Advance directive, Germany, Poland, Sweden

## Abstract

**Background:**

Healthcare legislation in European countries is similar in many respects. Most importantly, the framework of informed consent determines that physicians have the duty to provide detailed information about available therapeutic options and that patients have the right to refuse measures that contradict their personal values. However, when it comes to end-of-life decision-making a number of differences exist in the more specific regulations of individual countries. These differences and how they might nevertheless impact patient’s choices will be addressed in the current debate.

**Main text:**

In this article we show how the legal and medical frameworks of Germany, Poland and Sweden differ with regard to end-of-life decisions for patients with a fatal progressive disease. Taking Amyotrophic Lateral Sclerosis (ALS) as an example, we systematically compare clinical guidelines and healthcare law, pointing out the country-specific differences most relevant for existential decision-making. A fictional case report discusses the implications of these differences for a patient with ALS living in either of the three countries. Patients with ALS in Germany, Poland and Sweden are confronted with a similar spectrum of treatment options. However, the analysis of the normative frameworks shows that the conditions for making existential decisions differ considerably in Germany, Poland and Sweden. Specifically, these differences concern (1) the legal status of advance directives, (2) the conditions under which life-sustaining therapies are started or withheld, and (3) the legal regulations on assisted dying.

**Conclusion:**

According to the presented data, regulations of terminating life-sustaining treatments and the framework of “informed consent” are quite differently understood and implemented in the legal setting of the three countries. It is possible, and even likely, that these differences in the legal and medical frameworks have a considerable influence on existential decisions of patients with ALS.

## Background

Healthcare legislation in European countries is similar in many respects. Most importantly, codified in healthcare and patient laws, the framework of informed consent essentially defines the relationship between physician and patient. This framework involves two complementary rules: first, the physician’s duty to inform the patient about treatment options; and second, the patient’s right to give or deny consent to any therapeutic intervention the physician has offered [1]. When it comes to end-of-life decision-making, however, a number of critical differences exist in the more specific healthcare regulations of individual countries.

This article addresses the question how the legal and medical frameworks of Germany, Poland and Sweden differ with regard to end-of-life decisions for patients with a fatal progressive disease. Taking Amyotrophic Lateral Sclerosis (ALS) as an example, it compares healthcare law and clinical guidelines, pointing out the country-specific differences most relevant for existential decision-making, i.e. decisions to prolong or shorten life.

ALS is the most common adult-onset motor neuron disease. It is an invariably fatal medical condition, with a progressive generalized loss of voluntary muscle functions, including the ability to move, write, speak, chew and swallow. There is no cure and medical treatment is restricted to symptom control and palliative care - with ventilator support and enteral nutrition being among the most critical life-sustaining treatments (Table 1). In the final stage, many patients experience a locked-in state, with complete immobility (“quadriplegia”) and no ability to verbally communicate but an apparently clear mind (“deefferentation”). In the classic locked-in state there is sustained eye control. Varying degrees of cognitive and behavioral frontal lobe dysfunction are observed in as many as 20–48% of all patients, and 3–5% develop fulminant frontotemporal dementia (FTD). No effective treatment exists, and the median survival time is three to 4 years after onset. Patients usually die from either aspiration pneumonia or hypercapnia caused by respiratory insufficiency.

**Table 1:** Essential characteristics of Amyotrophic Lateral Sclerosis (ALS)

• Unfavorable prognosis	
• Survival time after onset 3–4 years	
• Progressive loss of voluntary muscle functions	
• In the course of the disease, most patients suffer from	
➢ dyspnoe requiring ventilatory treatment	
➢ dysphagia requiring enteral nutrition	
• Severe cognitive and behavioral impairment manifested as frontotemporal dementia (FTD) among 3–5% of patients	

While struggling with the disease’s impact on nearly all aspects of their lives, patients are confronted with a number of difficult medical choices. Most importantly, they have to make decisions for or against life-sustaining treatment measures. Based on the medical literature concerning ALS, we identify three distinct problematic situations in which existential decisions must be made: (1) communicating treatment options and advance care planning, (2) withholding or implementing life-sustaining therapies, and (3) continuing or withdrawing life-sustaining measures. These situations typically occur, respectively, in the early, advanced, and terminal stages of the disease [2–4]. In some countries, excluding the countries analyzed here, there is a fourth option of hastened death by means of active drug application to terminate life. However, this option is not further addressed as it is illegal in the hereby described context.

Decisions in these situations are mainly based on the individual patient’s clinical condition and personal values. However, individual choices are also shaped and constrained by a country-specific normative framework of legal regulations and clinical practices.

## Comparing the medical and legal frameworks for existential decisions in ALS

We compared the legal and medical frameworks regulating end-of-life decisions in Germany, Sweden, and Poland. The healthcare systems in these countries are quite similar. However, even slight variations in legal rights and clinical routines may have serious consequences for the medical pathway of an individual patient. In this article we identify to what extent the conditions for existential decision-making differ between the three countries under consideration. The potential effects of country-specific differences will be illustrated by referring to a typical clinical pathway of a patient with ALS.

Fictional case report:

*P.L. is a 56-year-old married machine operator and the father of two children. For 3 months, P.L. has experienced some weakness in his right hand, a general fatigability and slurred speech. He reports no cognitive difficulties. P.L. attends an outpatient clinic and is diagnosed with ALS. 7 months later, the symptoms have developed further: the weakness is more pronounced, and has extended to both arms and legs. His speech is less intelligible, and he has difficulty swallowing and breathing.*

*Due to the fast progression of symptoms, a number of essential medical decisions must be made. Within the next few months, P.L. must decide about therapeutic interventions for symptomatic management, such as non-invasive ventilation (NIV) and enteral nutrition* via *percutaneous endoscopic gastrostomy (PEG) or, if available, radiologically inserted gastrotomy (RIG). These may improve his quality of life and extend his life expectancy. In the latter course, there is the option to choose invasive ventilation (IV)* via *tracheotomy, which might extend his life up to 15 years or even more. However, as his immobility progresses, with invasive ventilation treatment he will probably proceed towards a locked-in state with no means to verbally communicate or move his body. He may learn to communicate* via *an eye movement-controlled computer. However, if he loses eyegaze control in the final stage of ALS, experimental communication with a brain computer interface will be the only alternative.*

*If P.L. accepts the above-mentioned therapeutic interventions, he might later decide to have them turned off. With no therapeutic interventions, he will likely die in less than a year.*

### Communication about treatment options and advance-care planning

The communication of the diagnosis is the first occasion at which physicians are in the position to inform patients about available treatment options and future implications of their diagnosis. The 2012 guidelines for the treatment of ALS, edited by the European Federation of the Neurological Societies (EFNS), urge physicians to proceed cautiously and to judge on a case-by-case basis how much the patient wants to know about choices that must be made in later stages of the disease. The diagnosis itself should be “pursued as early as possible”. It should be communicated to the patient as soon as the most established criteria are met [5]. At a minimum, patients should be informed about the neuroprotective treatment with riluzole, since this step must be taken immediately. Other treatment options, like enteral nutrition and ventilation therapy, may be raised at a follow-up visit, but physicians may be forced to discuss these issues at diagnosis if the patient already shows signs of malnutrition and/or respiratory insufficiency.

Given the sometimes rapid progression of symptoms, including the risk of developing FTD [6], the guidelines advise physicians to discuss treatment options and initiate advance-care planning at an early stage when the patient’s decision-making capacity is not yet doubted [7]. A written advance directive is recommended as a guarantee that no treatment will be initiated against the patient’s will, following the principle of respect for patient self-determination.

#### Similarities in the German, polish, and Swedish frameworks

In Germany, Poland and Sweden alike, physicians are obligated to comprehensively inform their patients about the benefits and risks of a proposed treatment. Patients, however, are entitled to set limits on the amount of information they wish to receive. Their right “not to know” needs to be respected and documented by the physician with an annotation in the patient’s medical record. Concerning the second rule, the final decision on the implementation of a particular treatment rests with the patient. After having been adequately informed of the benefits and risks, patients in Germany, Poland and Sweden have the right to refuse treatments that are incompatible with their personal values – even if a treatment is likely to prolong their life. The physician is authorized to initiate a particular treatment only if a patient has given fully informed consent (apart from extreme emergency, when the patient’s wishes are unknown) [8–11].

#### Differences in the German, polish, and Swedish frameworks

Germany

In Germany, one of the first objectives of physician-patient consultations after diagnosis is to determine a treatment plan and agree on a therapeutic goal. The physician determines which goals might realistically be achieved, and presents them to the patient. The patient chooses from the range of possible goals, and can change his or her decision at any time. Since no causative treatment is available for ALS, the spectrum of possible treatment goals ranges from prolonging life as long as possible (maximum goal) to ensuring symptom control (minimum goal) while otherwise allowing the patient to die peacefully. The treatment goal is to be documented in the patient’s record; e.g., with a primarily palliative goal, some life-prolonging interventions might not be considered [12, 13].

The process of physician-patient consultation can include the preparation of an advance directive in which the patient is able to decide for or against treatment options involving concrete future situations. Advance directives are legal documents in their own right, and are formally independent of the consultation or established treatment contract with the physician. Accordingly, the German neurological guidelines on ALS mention two separate goals for the early communication between physician and patient: the determination of treatment goals on the one hand, and the drafting of an advance directive on the other [14].

Since 2009, advance directives are legally binding in Germany. The new Act provides legal certainty that the provisions laid down in the living will of a currently incapacitated patient are to be treated as their expressed will and not merely their presumed will. The statements are required to be written, and the provisions must unambiguously apply to a concrete situation at hand. The act also strengthens the legal status of surrogate decision-makers who have been appointed by a patient in a custodianship directive. If explicitly authorized by the patient in writing, the “attorney” (caregivers, relatives or any other surrogate appointed by the patients) can now approve or deny life-sustaining treatment. [15].

Poland

In Poland, it is not standard procedure to determine goals in a treatment plan at an early stage of the disease. Therapeutic decisions on PEG, NIV or IV are made prior to a presumable need for intervention, and are subsequently written in the patient’s medical documentation. Polish healthcare law and clinical practice do not allow for formal advance directives. Thus, there are presently no legally guaranteed ways in which patients can influence their treatment choices in advance.

The possibility for such *pro futuro* statements, however, has been widely debated by experts [16]. An initiative to install a permanent representative, i.e. a surrogate decision-maker for the patient, has been undertaken but has not yet entered the legislative process [17]. Currently, a legal guardian can be assigned to an incapacitated adult patient only through a court ruling.

Sweden

In Sweden, after receiving adequate information about the prognosis and possible treatment options, the patient draws up a treatment plan with the physician [18]. Besides this agreement, patients may draft an advance directive, determining their treatment choices for a situation in which they might not be able to communicate. These advance directives, however, are not legally binding.

With respect to a surrogate decision-maker, it is not yet regulated who should make decisions for incapacitated patients. If a patient’s preferences and values are unknown, the responsible healthcare personnel is to act in the best interest of the patient. In practice, medical decisions for such patients are made by healthcare professionals, who try to reconstruct the patient’s presumed will in discussion with his or her relatives. The final decision is made by the responsible physician.

#### Consequences for a patient with ALS

*In the exemplary case of P.L., the attending physician in Germany, Sweden and Poland alike would have to inform him as early as possible of his diagnosis and recommend the treatment with riluzole, explaining its effects and limitations. Following the guidelines, the physician would also encourage him to discuss his treatment options at a follow-up visit. If P.L. did not wish to discuss treatment options for the terminal stage, this wish would have to be respected and the discussion postponed in all three countries.*

*Apart from these similarities, clinical procedures would differ somewhat. In Germany and Sweden there would soon be consultations, concluding with an agreement on a treatment plan reflecting the wishes and priorities of the patient. In both countries, the physician might also suggest that the patient draft an advance directive. The binding force of this document would remain unclear in Sweden, though. Since the option of drafting an advance directive or determining future treatment choices soon after the diagnosis would not be available in Poland, the physician would repetitively discuss treatment options with P.L. at subsequent visits.*

### Withholding or implementing life-sustaining measures

Invasive mechanical ventilation (IV) has given rise to controversy in the medical literature [19]. While more than 30% of patients with ALS in Japan receive long-term mechanical ventilation, IV is used only rarely in European countries [20, 21]. Despite empirical evidence of its life-prolonging effect, IV is not routinely offered by physicians and is only rarely requested by patients with ALS.

This particular issue tackles the more general debate on the legitimacy of withholding or proposing life-sustaining therapies. On the one hand, the physician’s authority to limit treatment can be legitimated as an indispensable barrier to potentially irresponsable requests by patients for maximum therapy [22]. On the other hand, the patient’s right to actively participate in end-of-life decision-making is justified as a necessary safeguard against the potentially subjective judgement by physicians concerning the presumed quality of life [23, 24].

In the scenario in which the physician favors a more extensive treatment, patients have the right to refuse therapeutic options that would contradict their values; patient autonomy trumps the physician’s strive for beneficence. In the inverted scenario, in which the patient wishes to start a life-sustaining therapy while the physician questions its usefulness, the principle of patient autonomy comes into conflict with the physician’s therapeutic prerogative and commitment to nonmaleficence. There is no obvious solution to this conflict, and the country-specific frameworks resolve it in different ways.

These difficulties are reflected in the recommendations of the EFNS guidelines concerning the treatment of respiratory insufficiency. While there are clear medical criteria for the initiation of enteral nutrition, the decision for or against a specific respiratory treatment is presented as a more delicate matter. The guidelines state that there “is no clear evidence regarding the timing and criteria” for the use of NIV and IV. Awkward conflicts should be prevented through early advance-care planning “before respiratory complications occur” [5]. More specific recommendations make it clear that decisions on respiratory management depend not only on the patient’s preferences and values but also on particular external factors.

**Fig. 1:**
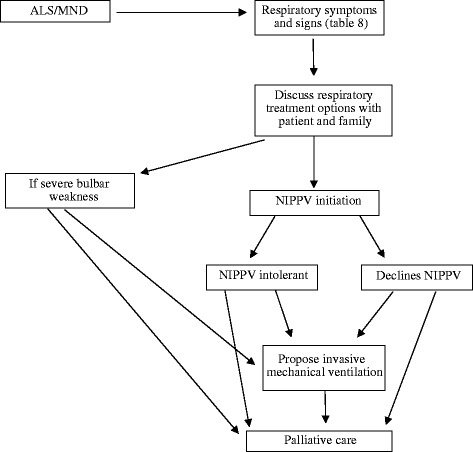
Flowchart for treating respiratory insufficiency according to EFNS guidelines

According to the guidelines, NIV is preferable to IV primarily because research has confirmed an improved quality of life through NIV, whereas for IV “no documented improvement in quality of life has been reported”. Although IV could prolong survival, “in some cases for many years”, it involved the “risk” that “some patients [would] develop a ‘locked-in’ state”. Second, the guidelines point to adverse economic and social implications of IV. It is “costly” and has “significant emotional and social impacts on patients and caregivers” [5]. Overall, IV is recommended only as a possible option rather than an obligatory therapeutic intervention (Fig. 1). The guidelines ultimately leave it open whether a physician should propose IV or recommend palliative care in the sense of basic comfort treatment for a dying patient. The accomodation of potentially conflicting preferences between physician and patient on this issue are thus left to country-specific regulations.

These recommendations are compatible with two different models of the physician-patient relationship. On the one hand, the reference to a patient’s advance directive and the openness to both IV and palliative care might point to a procedure of “shared decision-making”, in which the physician and the patient act as equals and share the responsibility for treatment choices [25–27]. The physician provides information about all potential treatment options in an unbiased way and then seeks an agreement with the patient on the preferred course of action. If we take a look at Emanuel and Emanuel’s “four models of the physician-patient relationship”, this approach corresponds to the “interpretive model”. According to this model, the physician takes the patients’ personal values and conceptions of a good life as the main benchmark for subsequent decisions. Because patients are not always fully aware of their values or of how they might relate to the treatment choices they confront, the physician has the duty of “helping to elucidate values and suggesting what medical interventions realize these values”. In reconstructing the patients’ goals and aspirations, the physician’s role is that of a well-intentioned “counselor, analogous to a cabinet minister’s advisory role to a head of state” [28].

On the other hand, the guidelines’ qualification of IV as optional and the accentuation of its drawbacks might point to a more hierarchical decision-making process in the physician-patient relationship, corresponding to Emanuel and Emanuel’s “deliberative model”. According to this model, the physician would help the patient to place isolated treatment decisions in a larger context, discussing the broader consequences of a treatment measure for the patients’ life. He or she would, however, focus on “health-related values” and assertively promote the medically indicated course of action. In contrast to the interpretive model, physicians would explicitly try to educate the patient in order to influence the decision-making process. As Emanuel and Emanuel make clear, “the physician aims at no more than moral persuation”. Acting as a “teacher or friend”, the physician “indicates what the patient should do, what decision regarding medical therapy would be admirable” [28].

Both these models fulfill the requirements of an “informed consent” framework, but differ in their approach to the communication between physician and patient.

#### Similarities in the German, polish, and Swedish frameworks

In Germany, Poland and Sweden, the constitution and fundamental legal provisions vest patients with the right of self-determination. Patients can choose between equally effective treatments; every therapeutic intervention requires their informed consent, and they are entitled to refuse options. Initiating a somatic treatment against the will of a competent patient is illegal. The patient’s right to make autonomous decisions hinges on three preconditions: patients can only approve or deny treatment options that the physician has actually proposed; they must be well informed; and they must be competent [11, 29].

If at this stage a patient is already showing clear signs of FTD, the physician must judge on an individual basis whether the patient is able to understand the implications of the respective intervention, and a surrogate decision-maker will be necessary. Legal guardians are normally appointed by a guardianship court, but German patients can also appoint them beforehand in their advance directive. This is not necessary in the case of moderate cognitive impairments as patients are still able to make autonomous decisions, which is clearly the case in the vast majority of ALS patients (> 90%) [30].

#### Differences in the German, polish, and Swedish frameworks

Germany

In Germany, physicians must propose only those therapeutic interventions they consider to be medically indicated. In established legal practice and in the juridical literature, medical indication is understood as a professional decision regarding the worthiness of treatment options in a concrete case. Analytically, it can be divided into two components. First, based on the likely prognosis and according to strict medical criteria the physician determines which treatment option would be suitable for attaining a stipulated treatment goal (“effectiveness”). The second component involves an evaluative judgement as to whether this treatment option is appropriate with regard to the situation and personality of a particular patient (“usefulness”) [31, 32]. A “sharing” of the criteria with the patient is possible, but not obligatory [13, 33].

In a verdict on 17 March 2003 the German Federal Court of Justice explicitly strengthened the role of the physician’s professional judgement in decisions concerning life-sustaining measures. It affirmed physicians’ authority to unilaterally withhold therapeutic options if they see a lack of medical indication [8]. According to the verdict, the patient’s right of self-determination can only be conceived as a “defensive right” against a particular treatment, rather than “as a claim to a particular treatment” [34]. In this sense, medical indication “circumscribes the area in which patient autonomy can unfold” [29].

Concerning the option of IV, the German neurological guidelines for the treatment of ALS follow the European guidelines on early counselling about the “prospects and limitations of non-invasive and invasive therapeutic options” [14]. The actual decision for or against the implementation of IV is clearly assigned to the physician. In line with the above-mentioned “interpretive model”, the guidelines recommend a “strict indication” with due regard for the “individual course of the disease” [14]. Similarly, the German guidelines for the treatment of respiratory insufficiency explain that tracheotomy is generally indicated when a non-invasive approach becomes ineffective, but further submit that “ethical concerns” such as the “loss of all arbitrary bodily functions in ALS” pose limits to its applicability [35]. Both guidelines take for granted that it is the task of the physician to determine whether the implementation of IV is appropriate in a specific case.

Still, the required reference to the individual treatment goal ensures that the patient’s preferences are duly taken into account [31]. For a patient with a primarily palliative treatment goal, withholding – and even not informing about – IV due to a lack of medical indication would be legally and professionally justified. By contrast, a patient with survival as the primary treatment goal would have to be comprehensively informed about IV when NIV fails. In this case, not discussing IV would amount to a change in the treatment goal, which can only be done after consultation with the patient [36].

In the last 10 to 15 years, there has been some controversy regarding the concept of medical indication, specifically raising the question whether it is adequate to understand it as a prerogative of the physician when it comes to end-of-life decisions [8, 37]. Attempts to push clinical practice towards the model of shared decision-making have proposed opening the process of medical indication and making its second component – the normative evaluation of usefulness – a regular matter of discussion between physician and patient [32, 33]. Clinical guidelines on end-of-life decision-making recommend an open-ended discussion about the benefit-harm ratio if the indication of a particular measure is “doubtful” and a “normative dissent” remains between the physician and a competent patient [38–40].

Poland

In Poland, it is also the physician who decides which treatment options are medically indicated for the individual patient. However, when it comes to the decision about life-sustaining treatment, the patient’s right to self-determination is somewhat stronger than in Germany. A physician in Poland is obligated to offer life-sustaining therapy if there is empirical evidence that it will increase a patient’s quality of life or prolong survival. An alert patient with ALS suffering from respiratory insufficiency would have to be offered the whole scope of effective treatments, in this case ranging from NIV and IV up to palliative symptom control. When a competent patient wishes to intensify treatment, the physician has the duty to provide it [41]. In case NIV is no longer efficient, the physician is obliged to propose IV to the patient, even if not being in favor of IV, and implement it at the patient’s request.

There is only one legitimate reason on the basis of which physicians in Poland are allowed to withhold life-sustaining therapies. According to the Code of Ethics, in terminal conditions a physician has no duty to provide reanimation, futile therapy or extraordinary measures [42–44]. In the case of an incapacitated patient, the physician should establish contact with the surrogate decision-maker. If this is not possible, the physician must consult another colleague before deciding whether a life-sustaining measure should be withheld [41].

Sweden

In Sweden, life-sustaining treatment has to be offered if there is a medical need for it; and a need is present if the measure benefits the individual patient. Both the National Policy Document of 1979 and the Patient Law state that life-sustaining treatments must be offered if they are indicated according to “science and proven experience” [11]. If the patient’s survival time or quality of life is not improved significantly by a particular measure, the physician decides whether it is indicated or should be regarded as “futile”. Furthermore, an indicated treatment must only be offered if the county – as the responsible healthcare authority – is willing to pay the costs. Even if the treatment might prolong a patient’s life, it could be regarded as too expensive by the authority [45]. For example, the county might refuse to pay for the supportive staff that is required for IV treatment if the patient is older than 65 years.

In contrast to some other countries, there is no official code of conduct regulating physicians’ actions, but there are soft laws and regulations specifying physicians’ duties. When it comes to end-of-life care, both the Swedish Medical Society and the National Board of Health and Welfare have provided guidelines [46–48]. The physician has a duty to inform and discuss end-of-life decisions with patients or, if they are not competent, with their surrogate decision-maker. Still, it is the physician who makes the final decision regarding which treatment is offered. Physicians are not legally obligated to offer life-sustaining treatment for which they see no medical indication, even if this might be against the wishes of a well informed, competent patient [49]. In the case of NIV ineffectiveness, for instance, the physician would decide whether to offer IV or palliative symptom control.

#### Consequences for a patient with ALS

*P.L. decides he wants to live as long as possible in order to take part in his children’s lives. Due to increasing symptoms of dysphagia, enteral nutrition is needed and a gastrostomy has to be instituted. Patient laws in Sweden, Germany and Poland alike would allow him to decide for himself between PEG or – if available – RIG.*

*As a consequence of his determination to live as long as possible, P.L. favors the maximum ventilatory support necessary for his survival. Based on the empirical evidence on patient satisfaction, the higher risk of infection in patients treated with IV, the considerably higher costs, and the need for 24-h professional nursing care, it is likely that the physician will first offer NIV. At the point at which NIV becomes ineffective, the question will arise as to whether the physician will offer IV.*

*Physicians in Germany ultimately have the authority to decide whether or not to propose a particular life-sustaining therapy. If P.L. had failed to clearly state his treatment goal, or if he had chosen a primarily palliative goal, it would be up to the physician to decide whether or not to inform him about and propose IV. But since P.L. has chosen the prolongation of life as his primary goal, the physician is obligated to inform him about this option. If the physician were nevertheless convinced that IV is not the appropriate option in P.L.’s case, he or she would be authorized to withhold IV due to a lack of medical indication. The next step could be to consult with the hospital’s ethics committee. If a consensus cannot be reached, the last solution might be a transfer to another physician.*

*In Poland, the situation for P.L. would be somewhat different. Here, as soon as he showed symptoms of respiratory insufficiency, the physician would have to inform him about all available treatment options: NIV, IV and palliative care. This would also be an appropriate time to inform him that, according to Polish law, once started an IV cannot be withdrawn even if the patient develops locked-in syndrome. After consultations with the physician, P.L. could autonomously decide if he wanted to be treated for respiratory insufficiency, and to what extent (NIV followed by palliative care or IV). Even if the physician were not in favor of IV in this particular case, P.L. could nevertheless demand its implementation once NIV no longer sufficed. The costs of the IV implementation and the patient’s management would be paid by the healthcare system at both the hospital and the home respiratory-care center.*

*Given that the Patient Law in Sweden explicitly demands the participation of patients in treatment decisions, it is likely that P.L. would be comprehensively informed about all available options, including IV. Swedish healthcare law also provides that it is the physician who decides whether a treatment is medically indicated. The physician might convey the criteria for a lack of indication,* e.g. *that the further progression of ALS would entail more suffering and less interaction than P.L. himself anticipates. P.L.’s chances of being offered IV would largely hinge on the professional opinion of the attending physician. Whether or not IV treatment could actually be initiated would also depend on the resources of the county and the willingness of the local authority to pay the necessary costs.*

### Withdrawing life-sustaining measures

While patients in Sweden, Germany and Poland have the right to refuse treatment options *before* they are initiated, their legal situation differs with regard to the elective termination of life-sustaining treatments *after* they have been started. Whether the discontinuation of ventilation therapy – most likely leading to the death of a patient with ALS – is allowed depends on the country-specific regulations regarding assisted dying and palliative sedation on request [50].

The EFNS guidelines do not address the legal or ethical preconditions for removing the ventilator of a patient with ALS. Recommendations are restricted to the factual statement that “parenteral morphine, a benzodiazepine and an antiemetic are used when the patient decides that ventilatory support should be withdrawn” to avoid dyspnoe, reduce anxiety and alleviate suffering [5]. Physicians discussing the withdrawal of the ventilator with their patients are medically and ethically obligated to offer accompanying sedation therapy [51, 52].

Two sedation procedures can be implemented for the withdrawal of ventilation therapy. First, if a mask-ventilated patient is still capable of residual spontaneous respiration, low doses of benzodiazepines and morphine are administered in order to achieve an “intensified symptom control”. This procedure also includes a high-dose oxygen therapy, leading to a narcosis by hypoventilation and carbon dioxide retention. Second, if the spontaneous respiration level is too low, and it is thus predictable that suffocation will occur immediately after the respirator is disconnected, “deep sedation” is recommended. In this case, the patient’s narcosis is induced by much higher doses of benzodiazepines and morphine [53].

The withdrawal of ventilator treatment raises further legal and ethical questions. Since the discontinuation of ventilator treatment is usually considered in the late stage of ALS, patients may be unable to communicate their wishes, or may have lost their decision competence due to advanced FTD. In such cases, the binding force of the patient’s advance directives, as well as the requirements for surrogate decision-making, needs to be reconsidered.

#### Similarities in the German, polish, and Swedish frameworks

Physician assisted suicide, also referred to as “active assisted dying”, is illegal in Germany, Poland, and Sweden alike. Unlike in Belgium, the Netherlands and Switzerland, physicians commit a criminal act if they administer a lethal dose of drugs with the intention of ending a patient’s life. The use of sedatives, however, is allowed if it primarily aims at the alleviation of suffering. Thus, physicians in all three countries are allowed to treat symptoms of respiratory insufficiency with benzodiazepines and morphine if these symptoms have proven to be refractory to other options. Concerning the withdrawal of life-sustaining measures, sometimes referred to as “passive assisted dying”, there are striking differences between the legal frameworks of the three countries. While withdrawing ventilatory treatment is permitted or even obligatory under certain conditions in Germany and Sweden, it is strictly forbidden in Poland.

#### Differences in the German, polish, and Swedish, frameworks

Germany

In 2010 the German Federal Court of Justice decided that life-sustaining treatment may be removed at the patient’s request even when death is not imminent. The Court argued that the right to self-determination allowed patients to change their treatment choices and revoke their earlier consent to a therapeutic measure. In the opinion of the Court, the “termination of treatment” was legitimate if it followed the “factual or presumed will of the patient”, and if it was primarily directed at “letting the progression of a disease take its course that without treatment would lead to death” [54].

In effect, withdrawing life-sustaining treatment is allowed and imperative if it corresponds to the patient’s will and/or if the physician determines a lack of medical indication [37, 55]. The ambiguous term “passive assisted dying” is now referred to either as “termination of treatment” or as “change in the treatment goal” [36]. The use of sedatives in the process of withdrawing ventilatory treatment (“palliative symptom relief”) is not only allowed but mandatory according to German healthcare law. In this case, the main purpose is to alleviate anxiety and prevent suffering. The potential shortening of the patient’s life is clearly an unintended adverse effect [55].

Thus, a competent patient with ALS is able to have ventilatory treatment withdrawn by changing the treatment goal [53, 56]. In the case of an incapacitated patient with a valid advance directive – stating, e.g., that ventilatory support should be withdrawn as soon as communication via eye movements is no longer possible – the physician would have to comply with the provisions of the living will. If patients are unable to communicate and have no valid advance directive for the current situation, their surrogate decision-maker is authorized to make this decision after consultation with the physician. Together, they reconstruct the presumed wish of the patient, referring to statements from earlier conversations concerning life in general as well as ethical and religious opinions [57, 58].

Decisions about life-sustaining treatments have to be revised on a regular basis. If the physician sees a lack of medical indication, or the treatment does not conform to the patient’s will, the physician has to consult with the surrogate decision-maker [59]. If both agree that termination of the treatment would correspond to the patient’s presumed will, ventilatory support can be withdrawn [13, 60]. The withdrawal of ventilatory support can be initiated by the responsible physician. If the parties cannot reach agreement, they have to consult the guardianship court [57].

Poland

In Poland, the withdrawal of life-sustaining therapy is illegal. There are no exceptions for what might be regarded as “passive assisted dying” [61]. Although the Medical Code of Ethics hypothetically allows for the termination of a treatment under certain conditions, the law leaves virtually no possibility to withdraw treatment. In cases in which ventilation has already been applied, the withdrawal of such therapy is considered an illegal act of assisted suicide to “be punished by imprisonment from 3 months to 5 years” [62]. Similarly, the Medical Code of Ethics, in Articles 30 and 31, prohibits euthanasia or physician-assisted suicide. Along these lines, the administration of opioids and benzodiazepines during the withdrawal of life-sustaining therapy would be regarded as an illegal act of “terminal sedation” [63].

Palliative sedation is only allowed for the purpose of symptom control in patients who are imminently dying [64], and can be used for a patient with respiratory insufficiency who does not want to undergo NIV or IV. Article 20 of the Act on Patients’ Rights and the Commissioner for Patients’ Rights guarantees the patient’s right to die in peace and dignity [65]. This includes “a right for medical services providing an alleviation of pain and other suffering” [41]. If the physician sedates a patient with the intention to alleviate suffering up to causing death, the act is considered “lethal analgesia” [63]. Ethical justification for lethal analgesia often invokes the principle of double effect – the acceptance of the fact of the foreseeable, but not intended, death of the patient [61, 66].

Sweden

In Sweden, the National Board of Health and Welfare published new recommendations in 2010 clarifying that withdrawing ventilator treatment, i.e. passive assisted dying, is allowed. Not granting the patient’s request is to be compared to somatic coercive treatment, which is prohibited according to the healthcare law and the constitution [67].

Sedation therapy is allowed. According to the guidelines of the Swedish Society of Medicine, it can be offered 14 days before expected death [47]. However, it is not clarified how it is possible to precisely estimate the remaining lifetime. The guidelines of the National Board of Health and Welfare do not state such time limitations; the only requirement is that there is a medical need, and this is the case if no other symptom relief is available. Both guidelines state that it is more important to alleviate a patient’s suffering, even though this might hasten death.

If the patient is no longer competent and the prognosis is fatal, it might be questioned whether ventilator treatment is still benefitting the patient. If a physician judges that invasive ventilation has become “futile” for a particular patient, soft laws and regulations have clarified that it is legally acceptable to stop treatment and therefore to let such a patient die peacefully. Professor of Law Madeleine Leijonhufvud has stated that if a patient is imminently dying, the duty to prolong his or her life becomes instead a duty to provide comfort care and the alleviation of all suffering. Treatments, apart from the comforting and alleviating ones, are no longer medically indicated [68], under the condition that the withdrawal of a treatment will not increase the patient’s suffering [67].

#### Consequences for a patient with ALS

*Having lived with invasive mechanical ventilation for several years, P.L. is exhausted from recurring infections and can now barely communicate with the eye-tracking device. He does not want to be a burden to his family, and requests that the respiratory treatment be terminated.*

*In Germany, if P.L. were still considered competent and explicitly expressed his will to withdraw IV, the physician would be allowed and obligated to follow his request. Also, if his competency were in doubt and he had clearly stated in his living will that the ventilator should be withdrawn as soon as he completely lost his ability to communicate, this decision would be legally binding. The physician would have to terminate ventilation therapy and provide for palliative sedation during the process of withdrawal.*

*Such a withdrawal of life-sustaining therapy would not be possible in Poland, for either competent or incapacitated patients. A physician would only be allowed to provide sedation therapy in a situation in which P.L.’s condition had deteriorated so greatly that death was imminent.*

*In Sweden, the physician would be allowed and obligated to remove the ventilator if P.L. were still regarded as competent, and if he directly requested this. If his mental capacity had deteriorated so greatly that he was declared incompetent, or if he were unable to express his will, the ventilator would not be allowed to be withdrawn, even if he had clearly stated in an advance directive that all life-sustaining treatment should be stopped under such conditions. Since advance directives are not legally binding in Sweden, P.L. would be at the mercy of the actions of his surrogate decision-maker and the judgement of the attending physician.*

## Conclusions

We have identified broad overlaps but also considerable differences in the German, Polish, and Swedish normative frameworks. Furthermore, we have spelled out their potential effects for the clinical pathway of a patient with ALS. In crucial situations, a patient like P.L. would make existential decisions in the context of country-specific legal regulations and clinical-medical routines. The main provision of the respective normative frameworks is presented in Table 2.

**Table 2:** Summary of country-specific conditions for existential decisions

	*Germany*	*Poland*	*Sweden*
Communication about treatment options and advance care planning	Physician informs the patient about treatment options and proposes a range of possible treatment goals		
	Patient and physician agree on a treatment plan and determine a treatment goal.	Agreement on a treatment plan and a treatment goal is not obligatory, and no standard practice.	Patient and physician agree on a treatment plan and determine a treatment goal.
	The patient may draft an advance directive, esp. a living will detailing his or her wishes concerning life-sustaining treatment, ideally, but not necessarily, after consultations with the physician.	Advance directives have no legal status in health care law.	The patient may draft an advance directive, esp. a living will detailing his or her wishes concerning life-sustaining treatment, ideally but not necessarily after consultations with the physician.
Withholding or implementing life-sustaining measures	The physician has the duty to inform about all life-sustaining measures that evidentially enhance quality of life and/or prolong life.		
	The physician proposes only those therapeutic measures that he/she considers to be medically indicated with regard to the treatment goal of the patient.	The physican proposes all available threapeutic measures that evidentially enhance quality of life and/or prolong life and that he/she considers to be medically indicated with regard to the disease stage.	The physician proposes only those therapeutic measures that he/she considers to be medically indicated for a particular need and that are approved by the clinic’s county.
	In the absence of a clearly stated treatment goal, the physician determines the medical indication of a life-sustaining measure in view of the concrete situation of the individual patient.		
	The patient can give consent or deny interventions proposed by the physician.		
		The patient decides which option is implemented. He/she can choose an intense and risky option even if it is not the one recommended by the physcian.	
Withdrawing life-sustaining measures	Withdrawal of life-sustaining measures accompanied by palliative sedation are allowed if:	Withdrawal of life-sustaining measures is regarded as an illegal act of “assisted suicide”.	Withdrawal of life-sustaining measures accompanied by palliative sedation are allowed if:
	1. a competent patient revokes the consent to continue a life-sustaining measure,		1. a competent patient revokes the consent to continue a life-sustaining measure,
	2. the physician determines a lack of medical indication due to the deteriorated condition of an incapacitated patient.		2. the physician determines a lack of medical indication due to the deteriorated condition of an incapacitated patient.
	Palliative sedation for symptom control and alleviation of suffering is allowed.	“Terminal sedation” accompanying the withdrawal of life-sustaining treatment is illegal. Palliative sedation for symptom control and alleviation of suffering is allowed for imminently dying patients (“lethal analgesia”).	Palliative sedation for symptom control and alleviation of suffering is allowed.
	In case of an incapacitated patient, the decision about the termination of treatment and palliative sedation has to conform to the living will of the patient or be made in agreement with the patient’s surrogate decision maker.	In case of an incapacitated patient, the decision for palliative sedation (“lethal analgesia”) has to be made in agreement with the patient’s surrogate decision maker.	In case of an incapacitated patient, the decision about the termination of treatment and palliative sedation has to conform to the living will of the patient or be made in agreement with the patient’s surrogate decision maker.

We conclude that patients with ALS in Germany, Poland and Sweden are confronted with a similar spectrum of treatment options. However, the decision of whether particular life-sustaining treatments are proposed or withdrawn is made in a country-specific context. Specifically, the legal and medical frameworks differ concerning (1) the legal status of advance directives, (2) the preconditions for implementing life-sustaining therapies, and (3) the regulations concerning the withdrawal of life-sustaining measures.

According to the presented data, the framework of “informed consent” – with the patient’s right of self-determination at its core - is quite differently understood and implemented in the legal setting of the three countries. It is possible, and even likely, that these differences have a substantial impact on existential decisions of patients with ALS.

## Abbreviations

ALS: Amyotrophic Lateral Sclerosis
EFNS: European Federation of the Neurological Societies
FTD: Frontotemporal Dementia
IV: Invasive Ventilation
NIV: Non-Invasive Ventilation
PEG: Percutaneous Endoscopic Gastrostomy
RIG: Radiologically Inserted Gastrotomy
